# Hospital Meetings

**Published:** 1907-03-16

**Authors:** 


### HOSPITAL MEETINGS.

### LONDON HOSPITAL.

A quarterly general meeting of Governors was held on Wednesday, March 6. The committee's report, after referring to the re-election of the Hon. Sydney Holland as chairman, and to the success attending the Christmas festivities at the hospital, went on to state that the grant from the King's Fund for last year was £8,500—£500 more than that for the previous year. The grant from the Hospital Saturday Fund was £1,162—practically the same as for 1905. The statistics with regard to the number of patients treated during 1906 showed that the demands on the hospital were greater than in any previous year. In 1906, 14,139 in-patients were treated, as compared with 13,552 in 1905; and the out-patients were 229,408, as compared with 209,272. The largest number of patients at one time in the wards was 835, and the average number of patients resident daily throughout the year was 790. In addition to the 13,429 in-patients treated last year, 185 children were born in the new "Marie Celeste" wards.

#### The Out-patient Work.

In the out-patient department the pressure of work was very great on the special departments; for instance, in the aural department 7,444 cases were treated; in the skin department, 6,933; and in the ophthalmic department, 7,496. Every possible care was taken to prevent any misuse of the hospital by people who could afford to pay a doctor. Some little inconvenience had been experienced owing to the large number of London County Council school children sent to the special departments. Under the existing laws the L.C.C. cannot treat children, but can only examine and advise them to obtain treatment. The ophthalmic and skin departments were accordingly inundated on certain days. A satisfactory arrangement has, however, now been made with the L.C.C. by which not more than fourteen children were to be sent to the hospital on any one day for treatment in a special department, and these only on four days a week. There has also been a great demand on the $x$-ray department. With the latest $x$-ray appliances a definite exposure could be given. This has been most useful in curing ringworm. Obdurate cases which had resisted other treatment for two or three years had been eventually cured by means of the $x$-rays. Many L.C.C. children had been treated by this method.

The income and expenditure account and balance-sheet for 1906 had not yet been completely examined by the auditors, but the following figures were available. The total ordinary income for the year was £76,509, and the income from legacies £12,922—together £89,431. The total ordinary expenditure was £93,291, and the extra-ordinary £30,057—together £123,348. It was satisfactory to note that annual subscriptions showed an increase of about £1,500 on the previous year, and donations an increase of £3,000.

The Nursing Staff.

The demands upon the private nursing staff during the year had been very great, and many applications had had to be refused. This was highly satisfactory as testifying to the efficiency of the nurses trained. The item extra-ordinary expenditure was heavy, but it was a matter of congratulation that the rebuilding of the hospital was entirely completed, and that the corresponding item for the current year would be extremely small. Next year would be the quinquennial appeal year, and there was every ground for expecting that the hospital would be able to replace certain capital, the sale of which had been necessitated by the rebuilding, which had cost altogether £470,000.

Hospital Legislation.

Since the last meeting of the Governors certain legislation affecting hospitals has had to be considered. Under the new Workmen's Compensation Act all nurses, probationers, and servants in the employment of the hospital fall within the scope of the Act in respect to accidents. It was at first thought that the hospital might also be liable in respect of diseases contracted by attendants from patients during the discharge of their duties. The hospital's solicitors were consulted, and gave it as their opinion that with respect to diseases the Act was directed against diseases known as industrial—*e.g.* phosphorus poisoning, and that the hospital would not be liable in this respect. The committee was, however, considering the question of the insurance of all nurses, probationers, and servants in order to cover all liability.

